# The “speed” of acuity in scotopic vs. photopic vision

**DOI:** 10.1007/s00417-020-04867-6

**Published:** 2020-08-15

**Authors:** Sven P. Heinrich, Torben Blechenberg, Christoph Reichel, Michael Bach

**Affiliations:** Eye Center, Medical Center – University of Freiburg, Faculty of Medicine, University of Freiburg, Killianstr. 5, 79106 Freiburg, Germany

**Keywords:** Visual acuity, Scotopic vision, Mesopic vision, Photopic vision, Temporal factors

## Abstract

**Purpose:**

The effect of duration of optotype presentation on visual acuity measures has been extensively studied under photopic conditions. However, systematic data on duration dependence of acuity values under mesopic and scotopic conditions is scarce, despite being highly relevant for many visual tasks including night driving, and for clinical diagnostic applications. The present study aims to address this void.

**Methods:**

We measured Landolt C acuity under photopic (90 cd/m^2^), mesopic (0.7 cd/m^2^), and scotopic (0.009 cd/m^2^) conditions for several optotype presentation durations ranging from 0.1 to 10 s using the Freiburg Acuity and Contrast Test. Two age groups were tested (young, 18–29 years, and older, 61–74 years).

**Results:**

As expected, under all luminance conditions, better acuity values were found for longer presentation durations. Photopic acuity in young participants decreased by about 0.25 log units from 0.1 to 10 s; mesopic vision mimicked the photopic visual behavior. Scotopic acuities depended more strongly on presentation duration (difference > 0.78 log units) than photopic values. There was no consistent pattern of correlation between luminance conditions across participants. We found a qualitative similarity between younger and older participants, despite higher variability among the latter and differences in absolute acuity: Photopic acuity difference (0.1 vs. 10 s) for the older participants was 0.19 log units, and scotopic difference was > 0.62 log units.

**Conclusion:**

Scotopic acuity is more susceptible to changes in stimulus duration than photopic vision, with considerable interindividual variability. The latter may reflect differences in aging and sub-clinical pathophysiological processes and might have consequences for visual performance during nocturnal activities such as driving at night. Acuity testing with briefly presented scotopic stimuli might increase the usefulness of acuity assessment for tracking of the health state of the visual system.

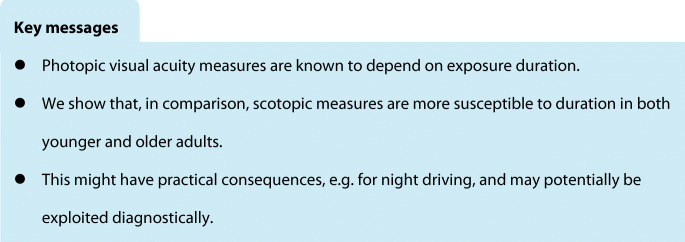

## Introduction

The duration during which each optotype is available for inspection during an acuity test affects the outcome of the test, with better values for longer durations [1–4]. This association is well established for very short durations where signal integration in the retina plays an important role [5, 6]. For longer durations, including those beyond 1 s, the association is less clear with some reports of acuity values that improve significantly [7, 8], while others report a non-significant trend [9] or no effect [6]. Several mechanisms may account for the prolonged duration effect, including variations in tear film [10], fluctuations in pupil size [11], accommodation [12], and cognitive factors [7].

While acuity testing is usually performed without strictly limiting the presentation duration, the dependence of the test outcome on the time available for inspecting the optotype has important implications. First, as tests are usually performed in a self-paced manner, the test outcome is likely to depend on how much time a person takes to look at an optotype and reach a decision. This means that personality and short-term changes in mental state play a role as a confounding factor in acuity testing. Second, vision in daily life may be very dynamic, for instance when driving. Prolonged inspection times in an acuity test may thus not be representative of real-world visual performance requirements. Third, temporal factors represent a key difference between subjective (behavioral) acuity testing and objective estimation of visual acuity, for instance by measuring visual evoked potentials [13, 14] or cognitive event-related potentials [15, 16]. Subjective methods are usually self-paced, and the examinee will normally evaluate the stimulus during an extended period of around 500 ms to several seconds. In contrast, the physiological responses on which the objective methods rely are determined by the initial few hundred milliseconds after stimulus onset. This difference may contribute to the shift of threshold in objective methods towards easier-to-recognize stimuli even when both methods, subjective and objective, use similar optotypes [16].

All abovementioned studies tested acuity under photopic conditions. A few also assessed conditions with lower luminance, albeit only covering a very limited parameter range [1] or restricted to mesopic conditions [17, 18]. Bartholomew et al. [19] found preliminary evidence for a duration effect under scotopic conditions. In their study, participants who took longer to respond to an optotype typically produced better acuity values under scotopic conditions. However, that study did not systematically investigate the influence of presentation duration.

The dependence of acuity on luminance has been of long-standing interest to researchers. Already in 1754, Mayer [20] (as cited in [21, 22]) noted that there is little effect of luminance on acuity within the normal photopic luminance range. A considerable number of studies followed over the next 200 years or so, which extended the range of interest to scotopic conditions, where visual acuity is considerably lower than under photopic conditions (e.g., [21, 23–28]).

The interaction of both aspects, luminance and presentation duration, may open up novel diagnostic approaches, given that on one hand, there are a number of visual impairments that are well known to be associated with reduced visual performance under mesopic and scotopic conditions [29–31], and on the other hand, there is evidence that various diseases have a strong impact on signal integration in the retina and on the temporal dynamics of perception, which may be exploited for diagnostic purposes [2]. Prospectively, better knowledge of the interrelationships between luminance and time will also help understanding the impact of specific visual impairments in situations with increased visual demand, such as nocturnal driving.

There is no single value that can be considered as the luminance level that segregates mesopic vision from scotopic vision. Rather, there is a complex interaction of several factors including stimulus size, eccentricity, and temporal parameters [32–34]. For the purpose of the present study, we will assume the mesopic range to extend down to 0.01 cd/m^2^, in agreement with previous studies (e.g., [35–37]).

The present study was designed to specifically assess the role of presentation duration under scotopic and mesopic conditions, and to compare the respective findings with the participants’ performance under standard photopic conditions. Furthermore, we assessed performance at additional intermediate presentation durations that were not included in our previous study on photopic acuity [7].

## Materials and methods

### Participants

The main group of participants consisted of 20 young participants (aged 18–29, 7 males and 13 females). Additionally, 10 older participants (aged 61–74, 5 males and 5 females) were tested. All participants had no known ophthalmological disorders, reached a corrected decimal acuity of logMAR = 0.1 or better in the study eye, and provided written informed consent. The study followed the tenets of the Declaration of Helsinki and belonged to a series of studies that was approved by the local institutional review board.

### Set-up

All testing was performed with the Freiburg Acuity and Contrast Test (FrACT) [38], version 3.9.8, available online: https://michaelbach.de/fract/. Landolt C stimuli were presented at a distance of 4 m on a TFT LCD monitor. The monitor’s front surface had a lightproof cover except for an aperture of approximately 15 × 15 cm. Multiple neutral density filters (Ningbo Haida Photo Supplies Co., Ltd., Ningbo, Zhejiang, China) could be inserted into this aperture to attenuate the light of the monitor. The set-up with a relatively small stimulus field of variable luminance in an otherwise dark environment approximates a situation at night with objects of different brightness.

For photopic testing, without filters, background luminance was 90 cd/m^2^. To yield scotopic luminance levels, two filters with nominal optical densities of 3 (1000×) and 0.9 (8×) were combined. For the mesopic condition, a single filter with a nominal optical density of 1.8 (64×) was used. The resulting luminance values were 0.009 cd/m^2^ for the scotopic stimuli and 0.7 cd/m^2^ for the mesopic stimuli, as computed from the unattenuated luminance of the monitor and the transmission of the filters. The latter was obtained from measurements of the luminance of a bright light source with and without the filters. These measurements were performed with both a Gossen Mavo Monitor (Gossen Foto- und Lichtmesstechnik GmbH, Nürnberg, Germany) and a Konica Minolta LS100 (Konica Minolta Co., Ltd., Osaka, Japan) and yielded values that were quite different from the nominal values for two of the filters (around 128× instead of 64× and 1200× instead of 1000×). All luminance values refer to photopic candelas.

Each test run consisted of 18 optotype presentations. A numerical keypad served as a response device. The FrACT uses an adaptive procedure to select the size of the next optotype, based on the history of optotype sizes and the corresponding responses in the respective test run. In separate runs, the duration of optotype presentation was set to 10 s, 3 s, 1 s, 0.3 s, and 0.1 s. Participants were instructed to not respond before the optotype had disappeared in order to take advantage of the full presentation duration.

### Procedure

Testing took place in a dark room behind an additional lightproof curtain. The monitor was the sole source of light. The participants were tested with their habitual correction in a single session, which started with a practice run with both eyes binocularly under normal luminance conditions to familiarize the participant with the test. Further testing was performed with only one eye, which was chosen ad libitum by the participant. The contralateral eye was covered.

The actual series of test runs started in the scotopic condition, followed by the mesopic and photopic conditions. Under each luminance condition, all 5 presentation durations were tested consecutively. The scotopic condition was preceded by 25 min of dark adaptation with the stimulus display already in scotopic configuration. Before the other luminance conditions, 2-min light-adaptation periods allowed the participants to adjust to the respective luminance increase. Between two test runs within a luminance condition, participants were given a 1-min rest.

### Analysis

All analysis was performed with Igor Pro (versions 7 and 8; Wavemetrics, Inc., Portland, OR, USA). We used resampling tests (50,000 samples) [39] for estimating statistical significance and computing confidence intervals in order to avoid relying on assumptions regarding the distribution of data. Because of the a priori hypothesis of logMAR values being larger (i.e., acuity being worse) for shorter presentation durations, one-sided tests were performed unless stated otherwise in the “Results” section.

## Results

### Main (young) group of participants

**Fig. 1:**
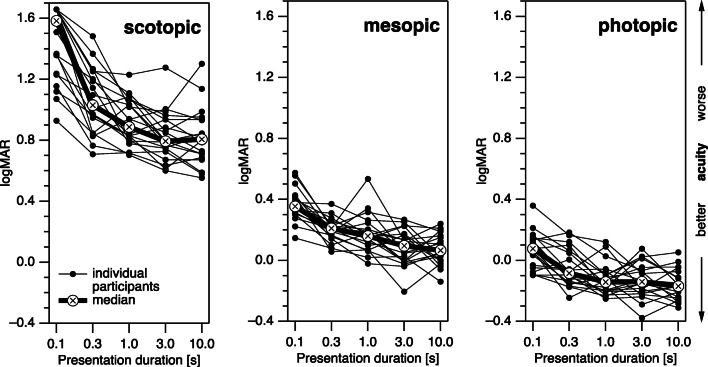
Results (logMAR) of individual young participants (thin lines) and respective median values (thick lines) for all three luminance conditions and all five presentation durations. For all luminance conditions, acuity values improved with both increasing presentation duration and increasing luminance. See text for display limitations of scotopic logMAR at 0.1 s

As illustrated in Fig. 1, acuity was generally best under photopic conditions, followed by mesopic conditions. Under scotopic conditions, acuity was worse by a sizable margin. Within each condition, median acuity improved with presentation duration (except for a slight contrary trend between 3 and 10-s duration under scotopic conditions). Importantly, with a duration of 0.1 s, threshold optotype sizes under scotopic conditions in several participants approached or exceeded the size of the aperture in the screen cover (15 cm ≙ 1.38 log arcmin). These values are therefore suffering from a ceiling effect.

The general increase in logMAR with shorter presentation durations was confirmed as highly significant by comparing the values at 10 s with the values at 0.3 s (*p* < 0.001, *p* < 0.001, *p* = 0.006 for scotopic, mesopic, and photopic stimuli, respectively, i.e., all significant at a family-wise *α* of 0.05). For exploratory purposes, the respective *p* values were also computed for comparisons between 10 and 1 s (*p* = 0.0035, *p* = 0.0006, *p* = 0.12) and 3-s presentation durations (*p* = 0.20, *p* = 0.22, *p* = 0.20).

**Fig. 2:**
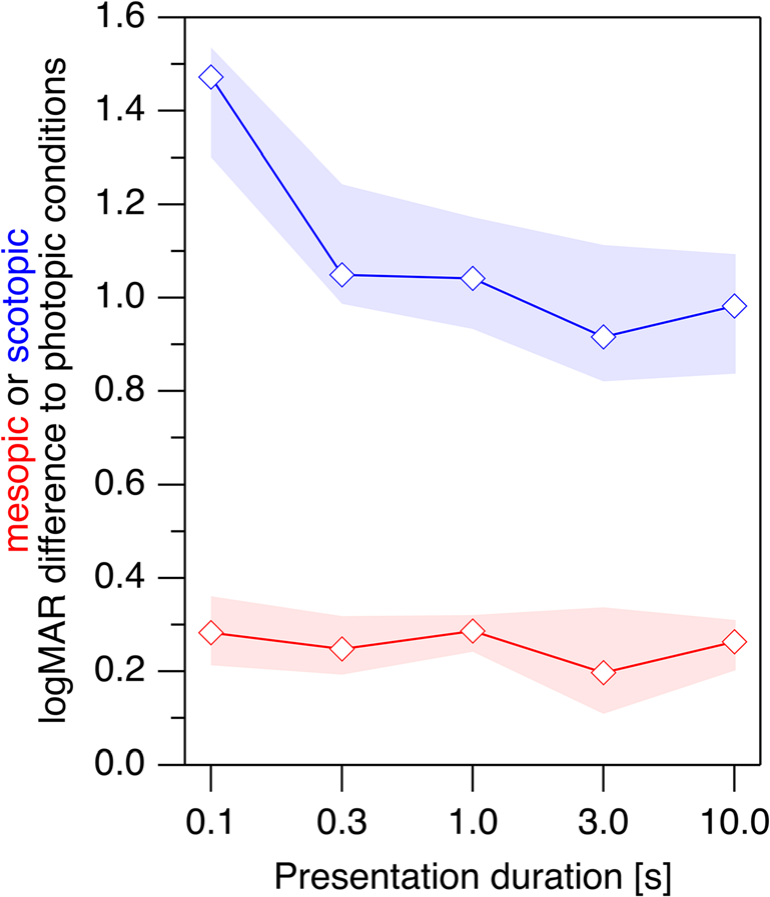
LogMAR differences between mesopic and photopic conditions (red) and between scotopic and photopic conditions (blue) of young participants. Shaded areas indicate the 95% confidence intervals. While there is no dependence on presentation duration for the mesopic vs. photopic logMAR difference, the scotopic vs. photopic logMAR differences are larger for the short presentation durations than for the long presentation durations. This suggests scotopic processing to involve additional integration processes

In order to assess whether the dependence on presentation duration differed between scotopic and photopic stimuli, we subtracted for each presentation duration the logMAR values of the photopic measurements from the respective values of the scotopic measurements (Fig. 2, blue trace). For statistical evaluation, we compared the 10-s value with the 0.3-s value (given that the 0.1-s value was not as trustworthy due to stimulus cropping and possible crowding as the threshold optotype size approached or exceeded the stimulus aperture size in several participants) via a two-sided permutation test, resulting in *p* = 0.0059. The corresponding comparison of the differences between mesopic and photopic conditions (Fig. 2, red trace) was not significant (*p* = 0.70).

**Fig. 3:**
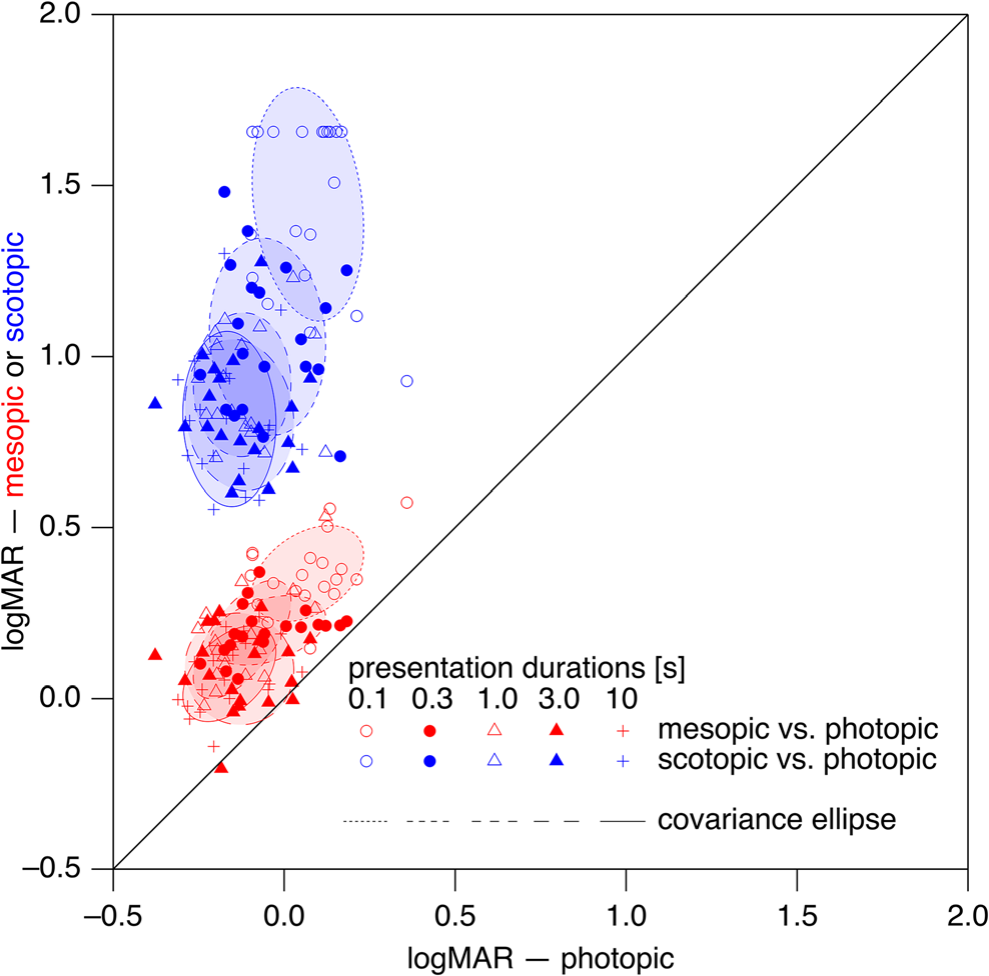
Scotopic (blue) and mesopic (red) logMAR values (ordinate) of young participants compared with photopic values (abscissa). The parametric covariance ellipses have been added to provide an approximate visualization of the data’s structure

Next, we tested whether logMAR under scotopic or mesopic conditions was correlated with logMAR under photopic condition, i.e., whether a participant with relatively good acuity under photopic conditions would also have relatively good acuity under scotopic conditions (Fig. 3). This was not the case for scotopic acuity (*p* > 0.5 for all presentation durations), and there was an inconsistent pattern for mesopic acuity with only some correlations being statistically significant on the single-test level (0.1 s, *p* = 0.15; 0.3 s, *p* = 0.0039; 1.0 s, *p* = 0.047; 3.0 s, *p* = 0.62; 10 s, *p* = 0.0066). With a Bonferroni-Holm correction [40] for 10 tests (5 mesopic and 5 scotopic), only one correlation (0.3 s, mesopic vs. photopic) was significant.

### Older participants

**Fig. 4:**
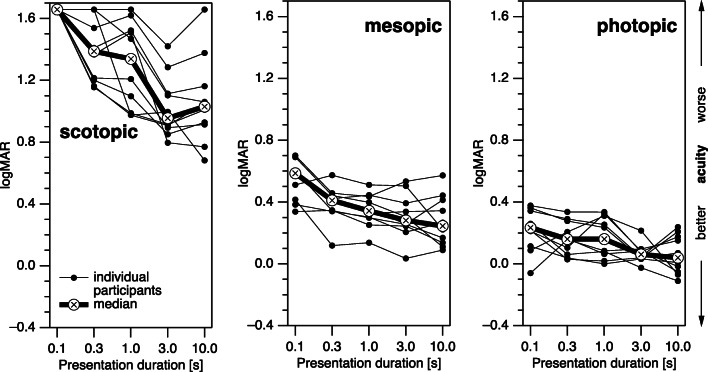
Results (logMAR) of individual participants above 60 years of age (thin lines) and respective median values (thick lines) for all three luminance conditions and all five presentation durations. For all luminance conditions, acuity values improved with both increasing presentation duration and increasing luminance. See text for display limitations of scotopic logMAR at 0.1 s

**Fig. 5:**
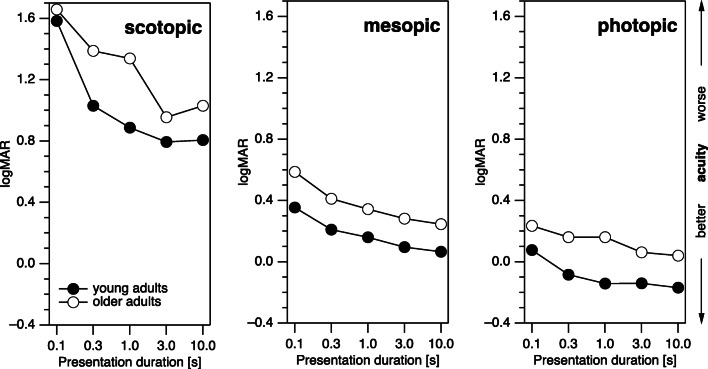
Comparison of median logMAR values of both young (open symbols) and older (filled symbols) participants. While acuity was generally better for young participants (i.e., logMAR values were lower), the dependence on presentation duration was similar

The older participants (Fig. 4) had somewhat higher logMAR values (worse acuity) than the younger participants (10-s duration, photopic, *p* < 0.001; mesopic, *p* < 0.0010; scotopic, *p = 0*.0017; Fig. 5). As with the younger participants, there was a significant effect of presentation duration (10 vs. 0.3 s, photopic, *p* = 0.015; mesopic, *p* = 0.023; scotopic, *p* = 0.016; all significant at a family-wise *α* of 0.05 with a Bonferroni-Holm correction [40]). The outcome of the statistical assessment did not change substantially when we removed from the scotopic data the one participant who had the logMAR values at ceiling for 4 out of 5 durations. Variability in the scotopic conditions appears higher in the older age group.

**Fig. 6:**
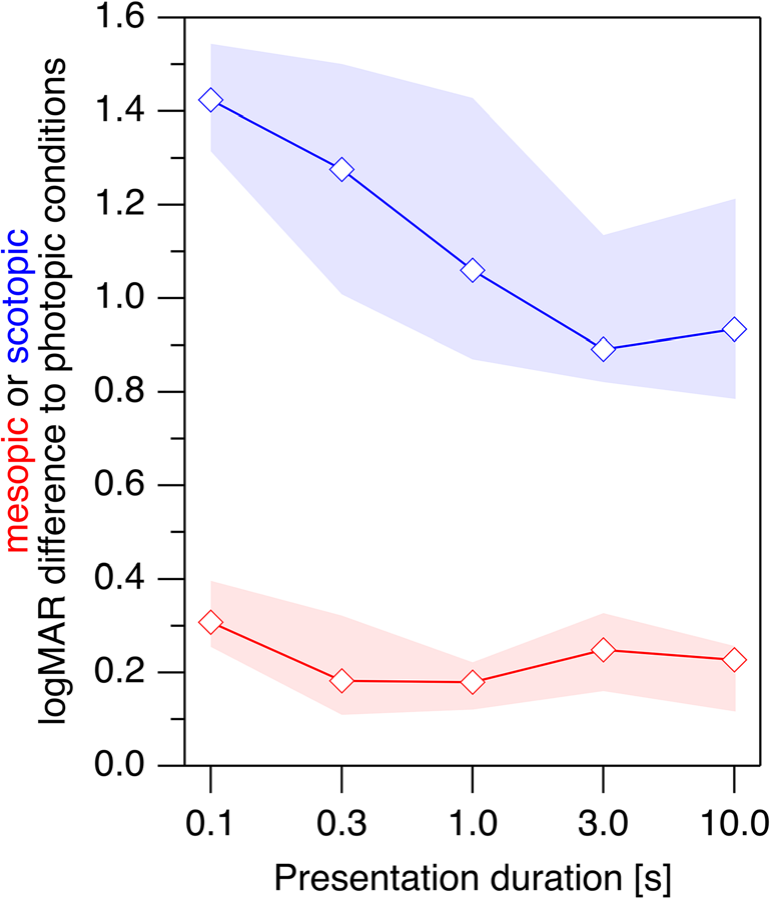
logMAR differences between mesopic and photopic conditions (red) and between scotopic and photopic conditions (blue) of participants above 60 years of age. Shaded areas indicate the 95% confidence intervals. While there is no sizable dependence on presentation duration for the mesopic vs. photopic logMAR difference, the scotopic vs. photopic logMAR differences is significantly larger for short presentation durations than for long durations

Following the same approach as with the younger participants, we also assessed in the older participants whether the dependence on presentation duration differed between scotopic and photopic stimuli. When subtracting for each presentation duration, the logMAR values of the photopic measurements from the respective values of the scotopic measurements (Fig. 6, blue trace), the comparison between the 10-s value and the 0.3-s value (given that the 0.1-s value was not trustworthy) was statistically significant (*p* = 0.0086.) The corresponding comparison of the differences between mesopic and photopic conditions (Fig. 6, red trace) was not significant (*p* = 0.46). We furthermore assessed whether the general logMAR difference between the mesopic or scotopic condition on the one hand and photopic condition on the other hand depended on the age group. This was not the case (10 s, mesopic, *p* = 0.38; scotopic, *p* = 0.42).

## Discussion

The main findings of the present study are as follows.

1. Unsurprisingly, under all luminance conditions, visual acuity improved with longer presentation durations.
2. Relative to photopic vision, scotopic visual acuity has a steeper dependence on presentation duration. Mesopic vision behaved similar to photopic vision.
3. There was no consistent pattern between presentation durations with respect to the correlation of acuity values between luminance conditions across participants.
4. Findings in the group of older participants were generally similar to those in the younger group, with at slightly worse acuity throughout.

### General difference between scotopic and photopic acuity

The logMAR difference between photopic and scotopic performance was about 1.0, i.e., a factor of 10 in terms of decimal acuity. This is less than the factor of around 15 found by Brown [41] (estimated from their figures). However, it is of a similar order of magnitude as the difference found by Shlaer [42], König [23], and Roelofs and Zeeman [25]. It also agrees well with recent data by Freundlieb et al. [43]. A large part of the resolution difference between scotopic and photopic vision is commonly attributed to post-receptor spatial integration [44].

### Effect of presentation duration

The present photopic data is in qualitative agreement with previous studies (e.g., [1–4, 9]). The logMAR decrease associated with increasing the presentation duration from 1.0 to 10 s is smaller than in our previous study [7], but statistically compatible (difference between studies in logMAR decrease 0.058, 95% CI – 0.023...0.099). The difference between 0.1 and 1.0 s is of similar magnitude in both studies. A detailed quantitative comparison with the various previous studies is difficult due to the very diverse testing conditions which may contribute to different effect sizes. So far, studies that systematically vary potentially relevant parameters to characterize and quantify their interaction with the general presentation time effect are lacking.

Regarding scotopic performance, our findings are consistent with those by Bartholomew et al. [19] that better acuity is, on average, associated with longer response times in self-paced testing. However, their data suggests that a response time increase from 1 to 10 s is associated with an average logMAR increase of 0.5 (see their Figure 5), which is about twice the difference in our data. Bartholomew et al.’s photopic logMAR data did not show a sizable effect of response time (their Figure S2), which might be because their set-up could not measure logMAR values smaller than − 0.18, with an ensuing ceiling effect in their data.

There are some differences between the present study and Bartholomew et al.’s study [19]. These could have an effect on the study outcome. For instance, the monitor distance was much larger in the present study, reducing the effect of insufficient accommodation which may occur when stimuli are presented briefly at a relatively short distance. Pupil size may also differ between studies, considering that it is affected by viewing distance and by the overall retinal illumination (which depends not only on the background luminance of the stimulus, but also on the aperture size and the surround), which implies differences in optical aberrations [45].

Although the scotopic logMAR values could not be accurately measured in the 0.1-s condition in several participants due to the restricted size of the stimulus aperture (resulting in stimulus cropping and crowding), it is clear that they are larger than those in the 0.3-s-condition, because otherwise, the geometric limitations of the set-up would not have been exceeded.

### Relationship between photopic and scotopic logMAR values

The pattern of correlations between photopic and scotopic logMAR values across participants was inconsistent between presentation durations. A few correlations reached statistical significance, albeit without a clear pattern. In particular, the lack of a significant correlation between scotopic and photopic performance for presentation durations of 1 s, 3 s, and 10 s agrees well with the non-significant correlation found by Freundlieb et al. [43] (see their supplementary figure) for self-paced tests. Bartholomew et al. [19] reported a significant correlation. However, this was with a very large number of participants and photopic acuity explained only 4.1% of the variance of scotopic acuity. The inconsistent pattern of correlation between mesopic and photopic logMAR reminds of the finding by Hertenstein et al. [46] of a partial dissociation between photopic and mesopic contrast sensitivity.

### Differences between young and older participants

It is well known that both photopic and, even more so, scotopic light sensitivity decrease with age [47]. It is thus not surprising that visual performance shows a general decline with age, although different visual tasks may be affected differentially in an interindividually variable manner [48]. In the present study, results in both age groups agree insofar as mesopic acuity shows a similar dependence on stimulus duration as photopic acuity, while scotopic acuity exhibits a higher sensitivity to presentation duration.

Informal exploration of the present data shows some features that may warrant further, more targeted, investigation in a future study. For instance, the strongest decrease in measured acuity (increase in logMAR) occurred when reducing presentation duration from 1.0 to 0.3 s in the older group, as opposed to the younger group where the strongest increase was between 0.3 and 0.1 s. Furthermore, interindividual variability among the older participants appears to be much more pronounced under scotopic conditions than under other luminance conditions, and also exceeds variability among young participants. This may be a correlate of different degrees of aging or of early signs of visual impairments that do not manifest (yet) in standard clinical tests. Combining scotopic luminance levels with short presentation durations may further enhance the diagnostic power of acuity testing.

In summary, the present study reveals a stronger dependence of scotopic acuity on presentation duration, compared with photopic acuity. However, there is considerable interindividual variability that may prove elucidative as to health and aging of a person’s visual system, and possibly fitness to drive at night.
